# Supplementary material for: Genomic landscape of pathogenic mutation of APC, KRAS, TP53, PIK3CA, and MLH1 in Indonesian colorectal cancer
Source: PLoS One. 2022 Jun 16;17(6):e0267090. doi: 10.1371/journal.pone.0267090 (PMC9202917; doi:10.1371/journal.pone.0267090)
Supplement: Attachment — Submitted filename: Response to Viewers.docx [file pone.0267090.s001.docx]

Reviewer #1: The article is the first study to analyze genomic profile of colorectal cancer patients in Indonesian population. The authors utilized an advanced genomics technology and identified mutations. The article is well written, and the data are sufficient to support the conclusion.
Minor things
[1] Typo in page 18 line 12: “underwent neoadjuvar ~” should be “underwent neoadjuvant ~”
[2] What is “Months” in the same page 18 line 12?

I already correct it to underwent neoadjuvant. The word “Months” is already deleted, and it was actually part of Figure 2. I already correct it as well in Figure 2. Thank you.

Reviewer #2: The authors have herein provided a snapshot of the mutagenic properties of colorectal cancer in a population of patients in Indonesia from 2017-2018.

This clinical synopsis is well-developed and interesting. Further, it is potentially applicable to future studies involving other populations as well as different cancer types, as a number of the predominant pathways and genes indicated here have been shown to play a role in a variety of malignancies across multiple populations.

Minor Revisions:

Page 25:
"(2) at least mutations in 4-5 genes are required for malignant formation; " - Please make this clearer for the reader. Are there specific genes you could identify that would frequently be mutated in order to induce disease, or does this simply refer to 4-5 random mutations?

“at least mutations in 4-5 genes are required for malignant formation”

I cited this sentence from Reference no.2 (Fearon ER, Vogelstein B. A Genetic Model for Colorectal Tumorigenesis. Cell. 1990;61:759-767).

The study explained that malignancy requires median of four to five allelic losses per tumor.

In earlier years, according to adenoma-carcinoma sequence theory (1990) by Vogelstein, only certain genes are known to be associated in each phase, but nowadays, with more advanced technology, more genes (rather than described in 1990) are involved.

So it is implicitly random, but saying random in this sentence seem inappropriate.

I add the word “any” 🡪 at least mutations in any 4-5 genes are required ……”

I hope it clears the confusion. Thank you very much.
