# Supplementary material for: Genomic landscape of pathogenic mutation of APC, KRAS, TP53, PIK3CA, and MLH1 in Indonesian colorectal cancer
Source: PLoS One. 2022 Jun 16;17(6):e0267090. doi: 10.1371/journal.pone.0267090 (PMC9202917; doi:10.1371/journal.pone.0267090)
Supplement: Attachment — Submitted filename: Response to Reviewers (2).docx [file pone.0267090.s002.docx]

Response to Reviewers

Additional Editor Comments:

1. Modify the original title "Indonesian Genomic Landscape of Pathogenic Mutation of APC, KRAS, TP53, PIK3CA, and MLH1 in Colorectal Cancer – How Different We Are from the World" to

"Genomic Landscape of Pathogenic Mutation of APC, KRAS, TP53, PIK3CA, and MLH1 in Indonesian Colorectal Cancer"

Already Changed – Thank you

2. Table 1: Stage 2A/B/C/      Number    5/1/-       Percentage should also follow similar format, i.e: 27/3/-.

Already changed – Thank you

3. All the gene names in: i) Title  ii) Table 2 iii) Table 3, 4, 9 iv) Figure legends and Table legends V) Manuscript text to be Italicized.

Already changed – Thank you

Including italicized ALL the gene names in the manuscript.

NB: but I did not highlight all the italicized gene
